# Integrating genetic, epigenetic, and clinical signatures via machine learning for robust prediction of leflunomide response in rheumatoid arthritis: a multi-center validation study

**DOI:** 10.3389/fimmu.2026.1804485

**Published:** 2026-06-24

**Authors:** Meng Chen, Haina Liu, Lei Jin, Xin Feng, Bingbing Dai, Fang Wang, Qiao Wang, Yulan Chen, Man Yi, Bowen Jia, Kangyi Dong, Jintao Zhang, Zhijun Fan, Jiahui Li, Feng Zhao, Yuanyuan Jia, Jianpeng Wang, Miao Liu, Jiayi Xu, Lingyu Fu

**Affiliations:** 1 Department of Clinical Epidemiology and Evidence-Based Medicine, the First Hospital of China Medical University, Shenyang, China; 2 Department of Rheumatology, The First Hospital of China Medical University, Shenyang, China; 3 Department of Rheumatology, ShengJing Hospital of China Medical University, Shenyang, China; 4 Department of Rheumatology, The First Affiliated Hospital of Jinzhou Medical University, Jinzhou, China; 5 Department of Rheumatology and Immunology, Central Hospital of Dalian University of Technology, Dalian, China

**Keywords:** DNA methylation, leflunomide, machine learning, rheumatoid arthritis, single nucleotide polymorphism

## Abstract

**Objective:**

To develop and validate a machine learning(ML)-based integrated predictive model combining genetic, epigenetic, and clinical factors for predicting leflunomide (LEF) treatment response in rheumatoid arthritis (RA) patients.

**Methods:**

A total of 357 RA patients (231 in the model development cohort [MDC], 126 in the external validation cohort [EVC]) were recruited from multiple centers in China. Whole-exome sequencing(WES), genome-wide DNA methylation profiling, and comprehensive clinical data were integrated for model development. Feature selection was performed via univariate analysis, Least Absolute Shrinkage and Selection Operator(LASSO) regression, and clinical feasibility filtering. Ten ML algorithms were tested, with SHapley Additive exPlanations (SHAP) for interpretability, and external validation to assess generalizability.

**Results:**

The final integrated model included 3 single nucleotide polymorphisms (SNPs: *ESR1-*rs2813563, *ABCC2*-rs4148396, *LMO4*-rs983332), 7 differentially methylated positions (DMPs: cg13568171-*MECR*, cg07694252-*ANGPT1*, cg13401893-*RNF39*, cg19814518-*UHMK1*, cg26370237-*HSF5*, cg11136343-*intergenic*, cg15961042-*intergenic*), and 3 clinical variables (IgG, course of disease, baseline Disease Activity Score 28(DAS28)). The Random Forest (RF) algorithm achieved the highest performance among the ten ML algorithms, with an area under the curve (AUC) of 0.84 (95% *CI*: 0.73–0.94) in the MDC and 0.70 (95% *CI*: 0.60–0.80) in the EVC—outperforming SNPs-only (AUC: 0.77/0.70) and DMPs-only (AUC:0.80/0.70) models. SHAP analysis identified cg13568171-*MECR* as the most important predictive feature, and its hypermethylation was associated with an improved response to LEF. Functional enrichment revealed three interconnected biological modules (lipid metabolism, drug transport, endocrine signaling) regulating LEF efficacy. A covariate-adjusted interaction between cg07694252-*ANGPT1* and *ESR1*-rs2813563 was observed in the MDC (*P* = 0.02).

**Conclusion:**

The integrated clinical-genetic/epigenetic RF model enables reliable prediction of LEF response in RA. Multi-omics integration showed superior performance in the MDC, while maintaining robust and non-inferior performance in EVC. The methylation-dependent interaction between cg07694252-*ANGPT1* and *ESR1*-rs2813563 implies a novel context-dependent transcriptional crosstalk mechanism, highlighting the value of multi-omics integration in advancing precision rheumatology.

## 1 Introduction

Rheumatoid arthritis (RA), a chronic systemic autoimmune disorder with a global prevalence of ~0.24% (1), inflicts substantial impairment on patients’ quality of life and imposes a heavy economic burden on healthcare systems worldwide. Despite major advances in RA therapy including conventional Disease-Modifying Antirheumatic Drugs (DMARDs) and targeted biologics (2, 3), treatment response heterogeneity remains a key unmet clinical challenge, highlighting the urgent need for personalized precision medicine strategies. LEF is a cornerstone of RA therapy with favorable efficacy and tolerability, and is generally regarded as having a comparable or more favorable safety profile relative to Methotrexate(MTX) in many clinical scenarios, especially in patients with MTX contraindications or intolerance, as supported by recent clinical guidelines and real−world evidence (4). LEF also offers substantial cost−effectiveness (4–6), yet approximately 30% of patients fail to attain an adequate therapeutic response (7). This unmet need underscores the critical demand for pretreatment predictive tools to optimize LEF administration and improve patient outcomes.

Existing LEF response studies have focused largely on single-layer biomarkers (8–11). Genetic variants affect LEF pharmacokinetics and safety (8, 9), and Han Chinese cohorts have confirmed relevant pharmacogenetic associations (10). Our previous epigenetics-only study identified predictive DMPs (11) but was limited by a single-omics design, small sample, and absence of external validation. Critically, the combined effects of genetics, epigenetics, and clinical factors—along with their interactions—remain poorly understood.

Genetic and epigenetic factors are key determinants of drug response, with SNPs at the forefront of biomarker research (12). Significant progress has been made in identifying genetic predictors for responses to MTX and Tumor Necrosis Factor-α (TNF-α) inhibitors [e.g., ATIC -rs4673993 and ABCB1-rs1045642 for MTX (13–15)]. To date, most ML-based prediction models for RA treatment response have focused on biologics or MTX, using single-omics or clinical data only. To the best of our knowledge, no ML-driven integrated model that combines genetic, epigenetic and clinical signatures has yet undergone external validation for LEF response in RA, nor has such a model been documented in prior publications. This gap is particularly striking given ML’s transformative impact on data-driven predictive modeling in healthcare (16): ML excels at integrating multi-omics and clinical data to decipher complex treatment response patterns, overcoming inherent limitations of traditional statistical methods (e.g., inability to capture non-linear relationships and high-dimensional interactions). Although ML has shown promise in predicting responses to various RA therapies, its application in optimizing LEF treatment remains largely unexplored—representing a critical missed opportunity to leverage advanced analytics for clinical benefit. Nevertheless, multi-omics integration coupled with ML also presents substantial challenges that must be addressed for reliable clinical translation. These include elevated risks of overfitting due to high-dimensional omics data, inherent “black-box” interpretability limitations of complex ML algorithms, and poor cross-cohort reproducibility stemming from population heterogeneity.

Notably, SNPs and Cytosine-phosphate-Guanine (CpG) methylation sites offer complementary biological insights: SNPs reflect inherent genetic variations that directly alter gene structure or function, while CpG methylation mediates dynamic epigenetic regulation of gene expression without changing the DNA sequence. Their integration enables a holistic exploration of LEF response mechanisms: It is biologically plausible that SNPs may influence transcription factor binding or the recruitment of epigenetic modifiers, thereby potentially shaping local methylation patterns. Similarly, CpG methylation may modulate chromatin accessibility and could potentially interact with SNPs to co-regulate gene expression and drug response. This synergistic approach captures complex molecular crosstalk, enhancing the robustness and clinical utility of predictive biomarker panels—addressing the key limitations of single-layer biomarker studies (including our prior work¹¹).

Against this backdrop, the current study aims to develop and externally validate an ML-based integrated predictive model combining genetic, epigenetic, and clinical factors to predict LEF outcomes in RA patients. We integrated WES for genetic SNPs, genome-wide DNA methylation profiling for epigenetic DMPs, and comprehensive clinical variables. Using a multi-center cohort of 357 patients (including a dedicated external validation cohort), we identified robust predictive biomarkers and built a clinically practical model. This approach overcomes key limitations of single-layer biomarker research, captures complex multi-omics interactions via ML, and provides new mechanistic insights into LEF response. Our model can support data-driven, personalized treatment decisions, improve therapeutic efficacy, and advance precision rheumatology.

## 2 Materials and methods

### 2.1 Study design and participants​

This multicenter prospective cohort study was conducted across four grade A tertiary hospitals in Liaoning Province, China, between June 2018 and June 2024. The study adopted a two-cohort design to ensure robust model development and external validation: (1) model development cohort (MDC) was constituted by a total of 231 RA patients from the two Affiliated Hospital of China Medical University, that is, the First and the Second (also called as Shengjing Hospital) Affiliated Hospital (Shenyang, Liaoning Province). (2) An independent external validation cohort (EVC) consisting of 126 RA patients was recruited from two geographically distinct institutions: the First Affiliated Hospital of Jinzhou Medical University (Jinzhou, Liaoning Province) and Dalian Central Hospital (Dalian, Liaoning Province), during the same time period with no overlap in patient recruitment or data collection with the MDC.

Inclusion Criteria:(1) Diagnosis of RA meeting either the 1987 American Rheumatism Association (ARA) revised criteria (17) or the 2010 American College of Rheumatology (ACR)/European League Against Rheumatism (EULAR) classification standards (18); (2) Aged ≥18 years, of Han ethnicity; (3) Initiation of continuous oral LEF therapy (20 mg daily) with a planned follow-up of ≥6 months; (4) Availability of complete baseline clinical, laboratory, genetic, and epigenetic data.

Exclusion Criteria: (1) Co-existing connective tissue diseases (e.g., systemic lupus erythematosus, scleroderma, Sjögren’s syndrome) or other inflammatory joint disorders (e.g., ankylosing spondylitis); (2) Severe comorbidities (e.g., malignant tumors, decompensated cardiovascular disease, end-stage renal/hepatic failure);(3) History of LEF allergy or contraindications to LEF treatment;(4) Incomplete follow-up data or loss to follow-up before 6 months.

Sample Size Calculation:According to methodological guidelines for ML–based predictive modeling, a minimum of 200 patients was required to achieve stable model development (19, 20). For external validation, the validation cohort was designed to be at least 50% the size of the development cohort to ensure a robust assessment of model generalizability (21, 22).

The study protocol was approved by the institutional review boards (IRBs) of all participating hospitals (ethical approval code: [2016]48), and written informed consent was obtained from each participant prior to enrollment. The study was conducted in accordance with the Declaration of Helsinki.

### 2.2 Procedures​

All enrolled patients received standardized LEF monotherapy (20 mg orally, once daily) for 6 months. All patients underwent a standardized 6-months follow-up period, with scheduled clinical visits at baseline (enrollment) and month 6 (final follow-up). Concomitant use of non-steroidal anti-inflammatory drugs (NSAIDs) and low-dose oral corticosteroids (≤10 mg/day prednisone equivalent) was permitted if clinically indicated (23, 24), with dosages recorded and adjusted uniformly across centers.

Data Collection:A centralized data management system was established to ensure consistency across centers. The following data were collected prospectively. (1) Demographic characteristics (age, gender, ethnicity, smoking/alcohol history); (2) Clinical parameters (course of disease, tender joint count (TJC28), swollen joint count (SJC28), visual analog scale (VAS) score for pain/fatigue); (3) Laboratory indices (erythrocyte sedimentation rate [ESR], C-reactive protein [CRP], immunoglobulin G [IgG], total cholesterol [TC], liver/kidney function); (4) Genetic/epigenetic data (SNP genotypes, CpG site methylation levels). Variables with a missing rate >20% were excluded, and values for variables with<5% missing data were imputed by chained equations.Laboratory tests were standardized with internal quality control across centers(**Supplementary Tables 1**, **2**). To ensure data consistency and minimize potential batch effects derived from multi-center recruitment and multi-omics profiling, all continuous clinical variables were z−score standardized to eliminate center−specific scaling differences. For genomic and DNA methylation data, platform−specific normalization and quality control pipelines were applied according to the manufacturer’s standard protocols.

### 2.3 Drug response assessment​

Treatment response was quantified using the change in DAS28 (ΔDAS28), calculated as the difference between baseline and six-month. DAS28 was calculated using the formula (25, 26):


$$\text{DAS}28=0.56\times \sqrt{\text{TJC}28}+0.28\times \sqrt{\text{SJC}28}+0.70\times \ln(\text{ESR})+0.014\times \text{VAS}$$

where TJC28 and SJC28 represent the counts of tender and swollen joints among 28 assessed joints, respectively. RA Patients were classified as responders if ΔDAS28 > 0.6 (indicating clinically meaningful improvement) and non-responders if ΔDAS28 ≤ 0.6 (27, 28). This definition is consistent with international RA treatment response criteria and ensures comparability with previous studies.

### 2.4 SNPs selection and genotyping​

Fasting venous blood was collected, and genomic DNA was extracted using a commercial kit. DNA quality was verified following standard protocols.

Candidate SNP Selection:A three-step strategy was used to identify 19 candidate SNPs:(1) WES of 3 responders and 3 non-responders (randomly selected) via multiplex PCR (Novogene Co., Ltd., Tianjin, China), with variants aligned to the GRCh37/hg19 reference genome and filtered by minor allele frequency (MAF) > 0.05; (2)Bioinformatics mining of public databases (Gene Expression Omnibus [GEO], Pharmacogenomics Knowledge Base [PharmGKB]) for SNPs associated with LEF metabolism or RA treatment response; (3)Text mining of PubMed/Embase using PubTator to extract literature-supported SNP-RA-LEF associations, Details are shown in **Supplementary Figure 1**.

Genotyping:Genotyping was performed on the Illumina HiSeq PE150 platform (Illumina, San Diego, USA) using high-throughput sequencing. Variant calling was conducted with SAMtools (v1.15), and genotypes were assigned using PLINK (v1.90). Samples with a call rate< 95% or Hardy-Weinberg equilibrium (HWE) *P* < 0.001 were excluded from analysis. Standard genotype calling and quality control procedures were applied to reduce technical variation, and no significant batch effects were observed after quality filtering.

### 2.5 CpG sites identification and methylation analysis​

Genome-wide DNA methylation profiling was performed using the Illumina HumanMethylationEPIC BeadChip (850K array) in 40 patients (20 responders and 20 matched non-responders) randomly selected from the MDC. DMPs were identified using thresholds of |Δβ|>0.1 and *P* < 0.05. To ensure independence and avoid overfitting, candidate CpG sites detected in this 40-sample nested subset were independently validated in the full MDC (n=231) and the EVC(n=126) using MethylTarget technology, with no overlap between the discovery panel and EVC. Methylation data were subjected to quantile normalization and strict quality control to minimize technical variation and batch effects across samples and runs.

### 2.6 Functional enrichment analysis​

Kyoto Encyclopedia of Genes and Genomes (KEGG) pathway enrichment analysis was performed for genes annotated to the identified SNPs and CpG sites. Gene function prediction and gene network analysis was performed by GeneMANIA online database (available at http://genemania.org/).

### 2.7 Statistical and ML analyses​

Statistical analyses were performed using R 4.2.0. Standard descriptive and comparative statistical methods were applied as appropriate.

To prevent data leakage, the MDC was randomly split into 70% training and 30% test subsets. A three-step feature selection procedure was applied exclusively to the training set: (1) univariate screening with *P* < 0.2; (2) 5-fold cross-validated LASSO for dimensionality reduction; (3) backward elimination to remove variables with negligible impact (ΔAUC<0.01). To improve stability, feature selection was repeated over 100 bootstrap iterations, and only consistently selected features were retained.

Model Construction and Evaluation:Ten ML algorithms were employed to construct predictive models integrating clinical and genetic/epigenetic features:(1)Ensemble methods: RF, Gradient Boosting (GB), Adaptive Boosting (AdaBoost);(2)Traditional statistical models: Logistic Regression, Linear Discriminant Analysis (LDA), LASSO Regression; (3) Other algorithms: Support Vector Machine with RBF kernel (SVM_Kernel), K-Nearest Neighbors (KNN), Partial Least Squares (PLS), Naive Bayes. At the study design stage, ensemble algorithms were predefined as core models, while other linear and conventional algorithms served as fixed baselines.

Model hyperparameters were optimized using grid search with 10-fold cross-validation. Model performance was evaluated using the following metrics: (1) Discriminative ability: AUC, sensitivity, specificity, precision, F1-score; (2) Calibration: Hosmer-Lemeshow test and calibration curves; (3) Clinical utility: decision curve analysis (DCA) and clinical impact curves (CICs).

Model Interpretation: SHAP analysis was performed to quantify the marginal contribution of each feature to model predictions and visualize feature-response relationships.

External Validation: The optimal model was validated in the independent EVC without any parameter adjustment.

## 3 Results

### 3.1 Demographic and clinicopathological characteristics​

A total of 412 RA patients were initially enrolled. During follow-up, 55 patients were excluded, leaving 357 patients for final analysis. Exclusions were as follows: (1) loss to follow-up (n=32, 58.2%), 28 (87.5%) of whom were lost due to COVID-19 pandemic-related restrictions (2020.01–2022.12); (2) voluntary withdrawal (n=14, 25.5%); (3) non-adherence to LEF therapy (n=9, 16.3%). No patients were excluded due to severe LEF-related adverse events.

Among the 357 included patients, 83 (23.25%) achieved remission and 47 (13.17%) achieved low disease activity, with a total of 130 patients (36.42%) attaining favorable clinical status. Details can be found in **Supplementary Table 6**.

Specifically, the MDC was constituted by 231 RA patients (follow-up completion rate: 83.7%). The EVC included 126 patients (follow-up completion rate: 92.6%). There was no significant difference in the follow-up completion rate between the two cohorts (χ² = 3.21, *P* = 0.074).

All the patients were classified as responders (n = 243, 68.1%) or non-responders (n = 114, 31.9%) based on ΔDAS28. Baseline characteristics, including age (MDC: 59.13 ± 13.34 years vs. EVC: 57.90 ± 10.15 years; *P* = 0.19), gender (female proportion: MDC 75.76% vs. EVC 81.75%; *P* = 0.24) showed no statistically significant differences between the MDC and EVC (in **Supplementary Table 3**). The comparable baseline profiles between cohorts and between response subgroups confirm the validity of subsequent model development and validation.

### 3.2 Screening of predictive features

**Figure 1:**
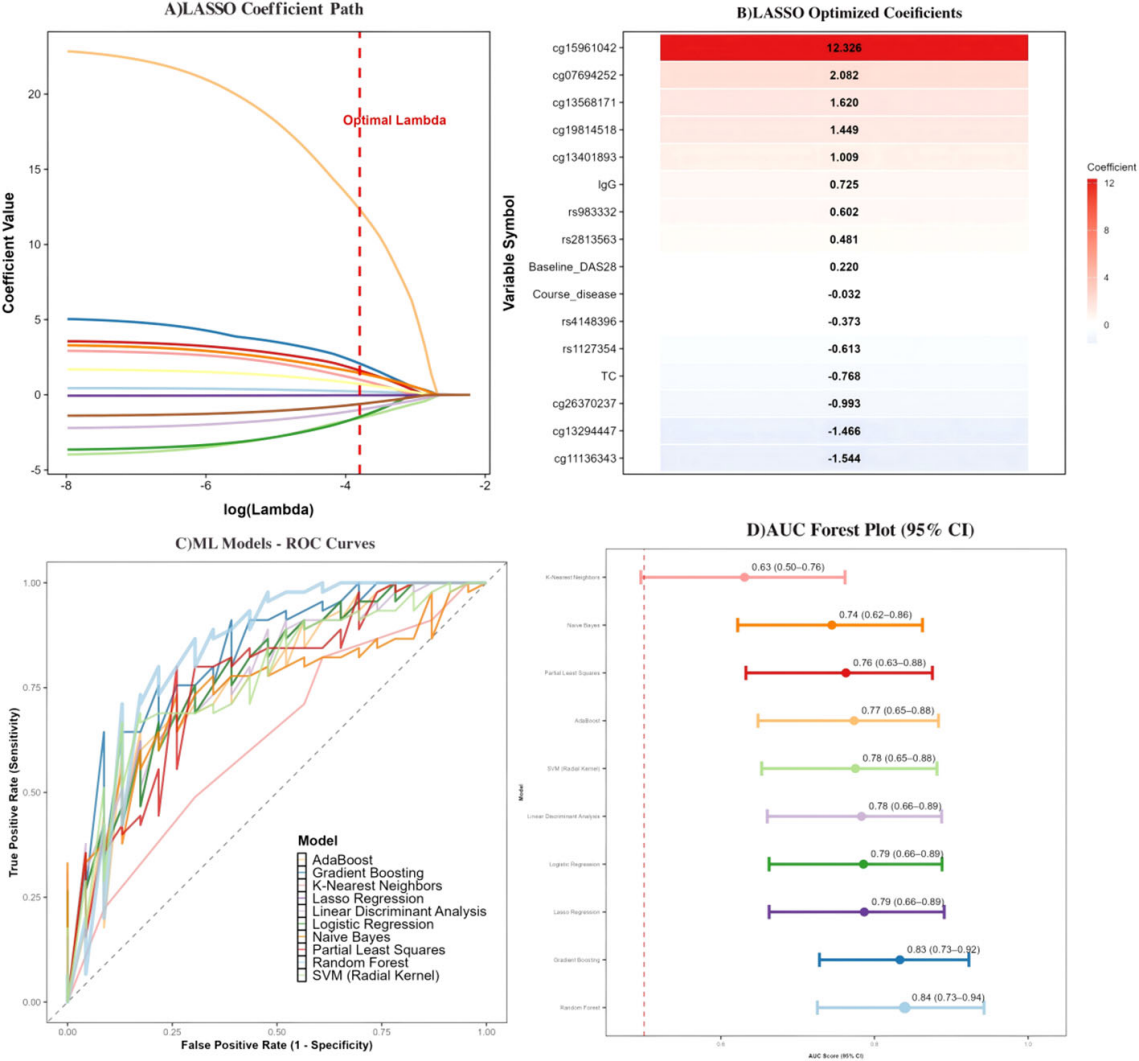
Variable selection and algorithm evaluation for the LEF response prediction model. **(A)** LASSO coefficient paths for variable selection. **(B)** Coefficients of selected variables in the optimized LASSO model. **(C)** ROC curves of 10 ML models for LEF response prediction. **(D)** Forest plot of AUC values (with 95% CIs) for the 10 models.

The initial high-dimensional feature set included 19 candidate SNPs, 63 whole-genome DNA methylation sites, and 31 clinical variables. During the feature variable selection process, 28 candidate variables were initially determined through univariate analysis (*P* < 0.2). Through LASSO regression (optimal lambda=0.022) and subsequent dimensionality reduction using 5-fold cross-validation, the candidate set was further reduced to 16 variables, as shown in **Figures 1A, B**. Considering practical clinical factors such as medical cost-effectiveness and accessibility, 3 variables (TC, *ITPA*-rs1127354, and cg13294447-*intergenic*) were eliminated using the backward elimination method. These variables had minimal impact on the model’s AUC (ΔAUC<0.01). Ultimately, the optimal model contained only 13 features. The ratio of features to samples in the model development cohort (n=231) was 1:17.8, and in the entire cohort (n=357), it was 1:27.5, significantly reducing the risk of model overfitting.

The final results revealed that IgG, course of disease, and baseline DAS28 were the most clinically relevant phenotypic predictors, ten robust genetic/epigenetic markers were identified: *LMO4*-rs983332, *ESR1*-rs2813563, *ABCC2*-rs4148396, cg13568171-*MECR*, cg07694252-*ANGPT1*, cg13401893-*RNF39*, cg19814518 -*UHMK1*, cg26370237-*HSF5*, cg11136343-*intergenic*, and cg15961042 -*intergenic*. These 13 features (3 clinical + 10 genetic/epigenetic) were integrated to construct a predictive model for predicting LEF treatment response in RA patients, with detailed specifications provided in **Supplementary Table 5**.

### 3.3 Algorithm selection and model evaluation

Model performance was systematically evaluated using sensitivity, specificity, AUC, F1-score, and precision for ten ML algorithms. As shown in **Supplementary Table 4**, the RF model outperformed all other algorithms. ROC curve analysis (**Figure 1C**) demonstrated that the RF and GB models consistently exhibited superior discriminative ability across different false positive rates compared to other models. Quantitative analysis of AUC scores in the validation cohort (**Figure 1D**) revealed that the RF model achieved the highest AUC of 0.84 (95% *CI*: 0.73–0.94), followed by the GB model with an AUC of 0.83 (95% *CI*: 0.72–0.93). Other models, including LASSO and Logistic Regression (both AUC = 0.79, 95% *CI*: 0.66–0.89), and LDA (AUC = 0.78, 95% *CI*: 0.67–0.89), also demonstrated moderate discriminative power, while the KNN model performed the least effectively (AUC = 0.63, 95% *CI*: 0.50–0.76).

**Figure 2:**
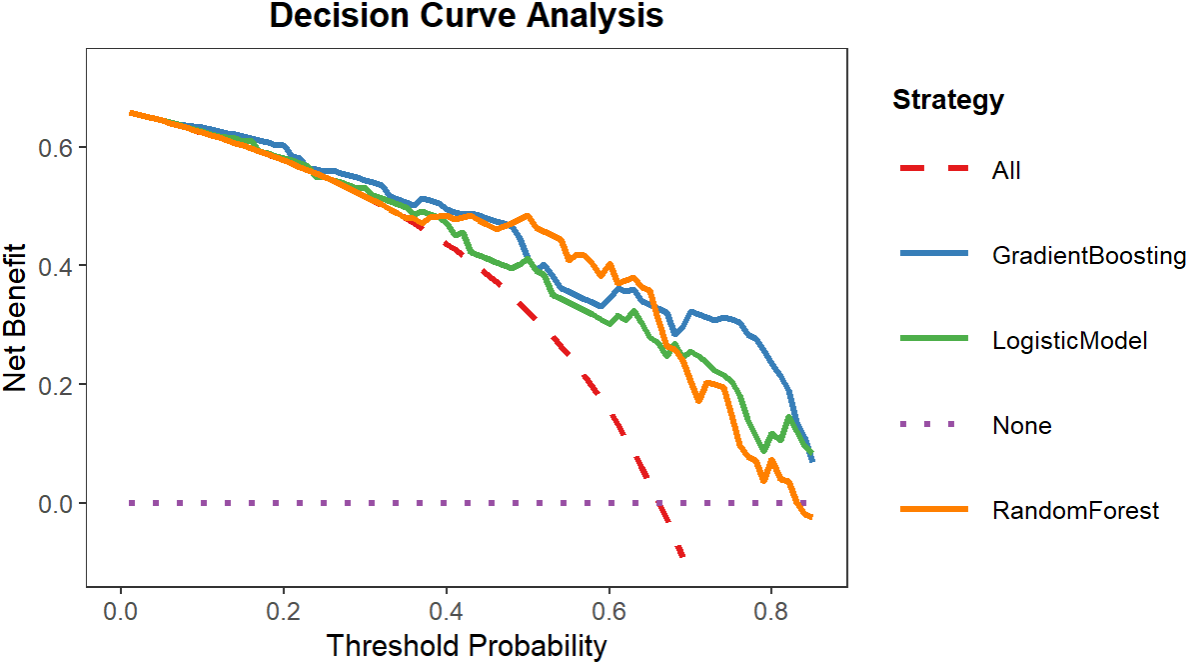
Decision curve analysis of the top-three models in MDC.

DCA (**Figure 2**) showed that the three best-performing models (GB, RF, Logistic Regression) achieved higher net benefit than “treat all” or “treat none” strategies across clinically relevant thresholds. Given its high AUC and comparable clinical utility, the RF model was selected as the optimal model for guiding LEF treatment decisions.

### 3.4 Interpretation, calibration, and clinical utility of the random forest model

**Figure 3:**
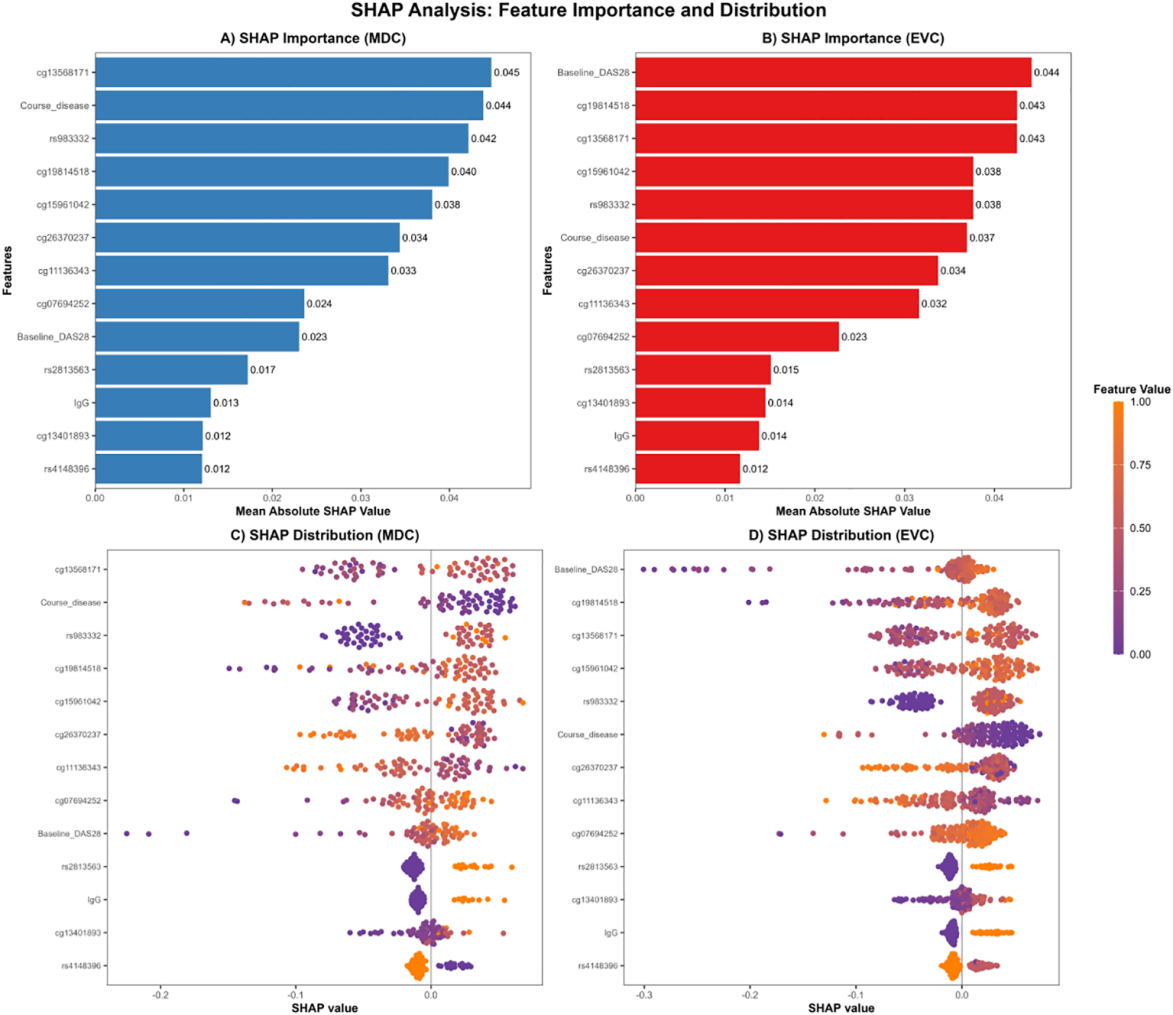
SHAP-based feature importance and distribution for the random forest LEF response model. **(A, B)** SHAP feature importance in the MDC and EVC datasets. **(C, D)** SHAP value distribution of features in the MDC and EVC datasets.

**Figure 4:**
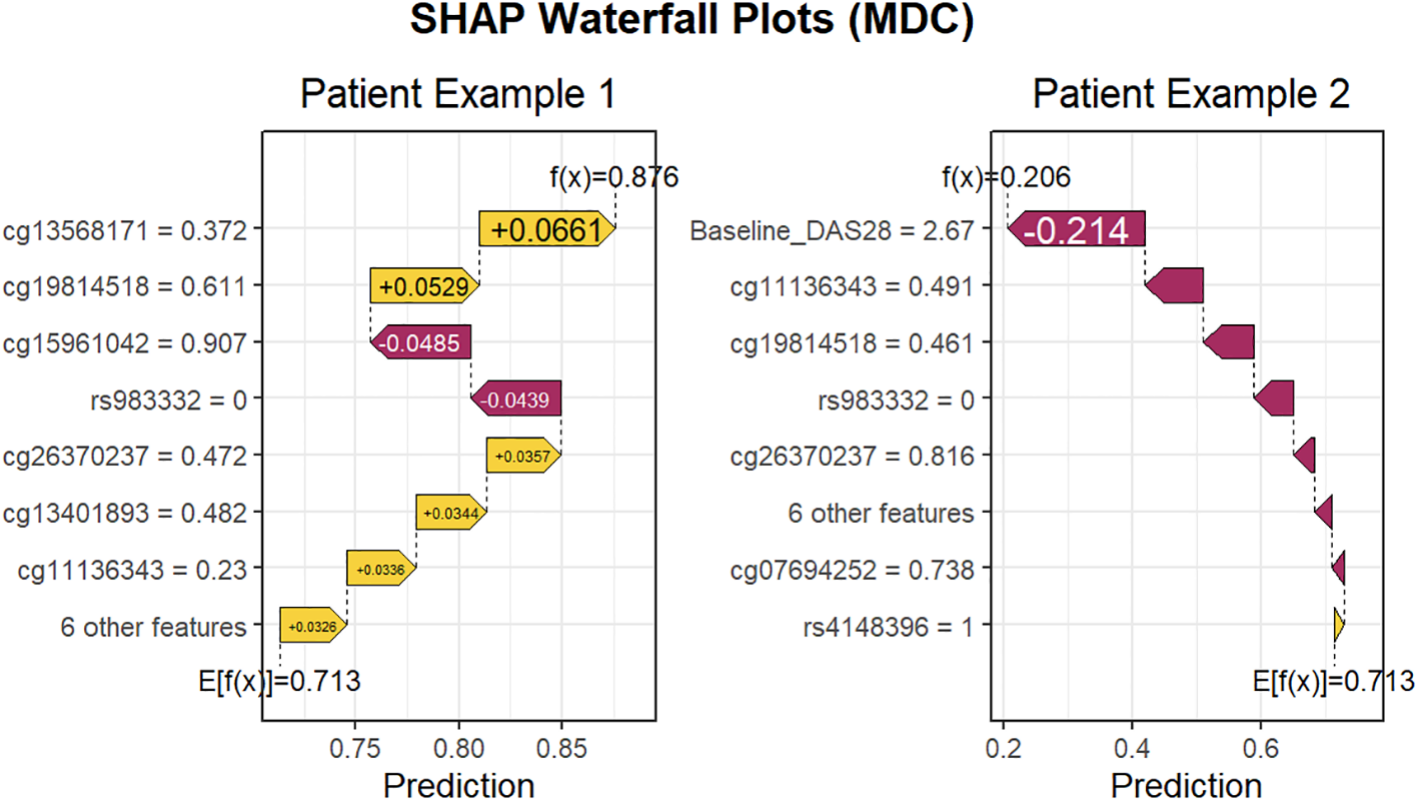
SHAP waterfall plots for individual patient-level model interpretation.

SHAP analysis (**Figure 3**) quantified feature contributions and identified cg13568171-*MECR* and disease course as the top predictors in the MDC, whereas baseline DAS28 was the most important in the EVC, indicating context-dependent importance. SHAP waterfall plots (**Figure 4**) further illustrated individual predictions: Patient 1 showed a high response probability (f(x)=0.88) driven mainly by cg13568171-*MECR* (+0.07), while Patient 2 had a low probability (f(x)=0.21) dominated by negative contributions including baseline DAS28 (−0.21).These findings support the biological plausibility of the multi-omics model.

**Figure 5:**
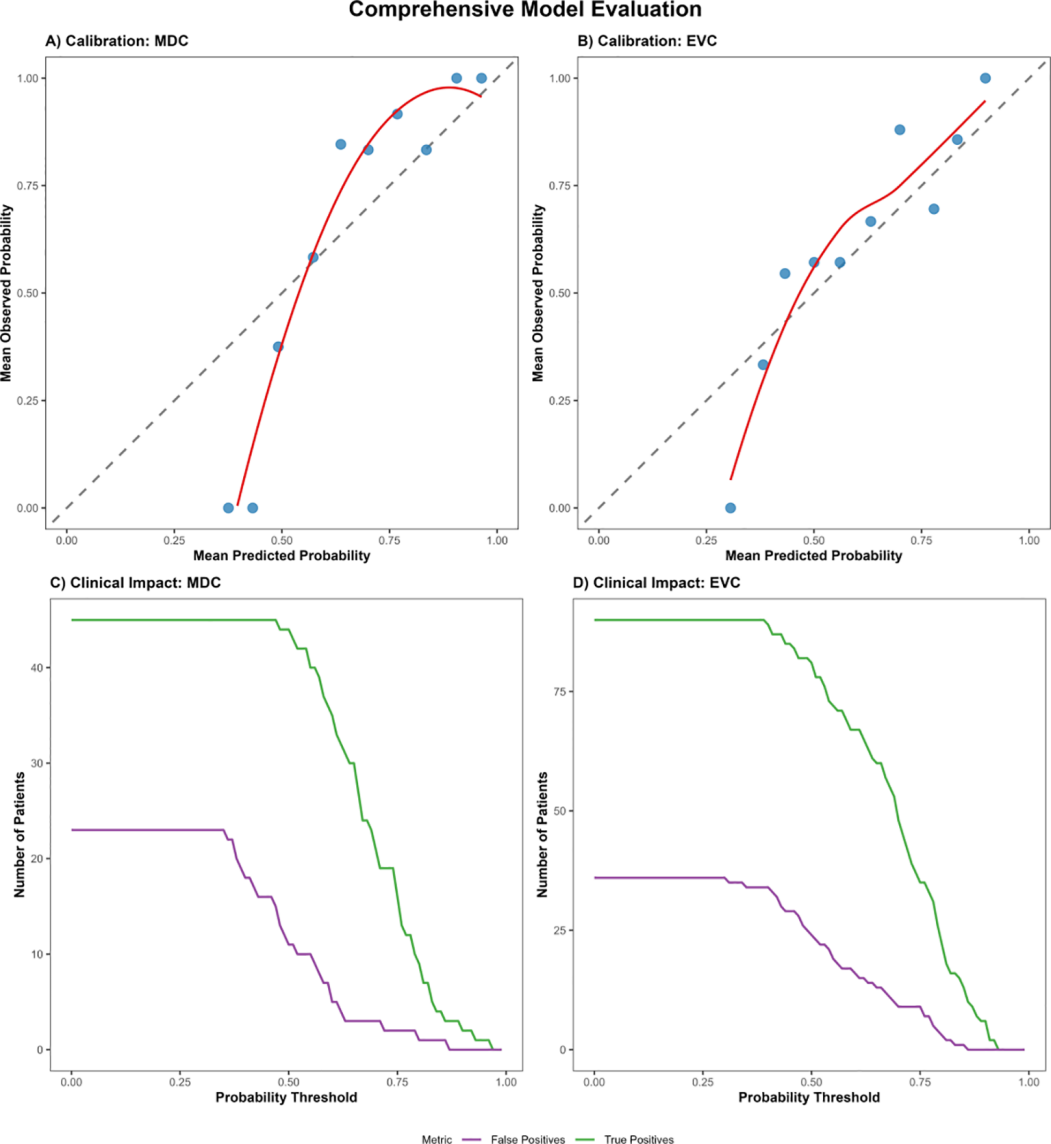
Performance evaluation of the random forest LEF response prediction model. **(A, B)** Calibration curves in the MDC and EVC datasets. **(C, D)** Clinical impact curves in the MDC and EVC datasets.

The calibration curves (**Figure 5**) assess the agreement between the RF model’s predicted probabilities and the observed outcomes in the MDC (**Figure 5A**) and EVC (**Figure 5B**). Both passed the Hosmer-Lemeshow test (MDC: *P* = 0.13; EVC: *P* = 0.62). In the MDC, the model demonstrates reasonable calibration overall, with the red curve closely aligning with the dashed reference line (representing perfect calibration) across most ranges of mean predicted probability, though there is some deviation at the extreme ends. In the EVC, the curve also shows a generally good fit to the reference line, indicating that the model maintains its calibration performance when applied to an independent cohort. This consistency in calibration across both sets suggests that the model’s predicted probabilities are reliable and can be interpreted with confidence in clinical or research settings, which is crucial for translational applications where accurate probability estimation informs decision-making.

The CIC curve (**Figure 5**) illustrate the number of true positives and false positives of the RF model across varying probability thresholds in the MDC (**Figure 5C**) and EVC (**Figure 5D**). In both cohorts, the green curves (true positives) remain relatively stable at lower probability thresholds, indicating the model can identify a large proportion of positive cases without excessive false positives, as evidenced by the flat purple curves (false positives) in the early threshold ranges. As the probability threshold increases, the number of true positives decreases gradually, while false positives decline sharply, demonstrating the model’s ability to balance true and false positive rates across different clinical decision thresholds. This pattern is consistent between the MDC and EVC, suggesting the model’s clinical utility is reproducible across independent cohorts. Such performance implies that the model can be tailored to different clinical scenarios—for instance, adopting a lower threshold to maximize case detection or a higher threshold to minimize false alarms—thereby supporting informed clinical decision-making in diverse settings.

### 3.5 External validation and generalizability of the RF model

**Figure 6:**
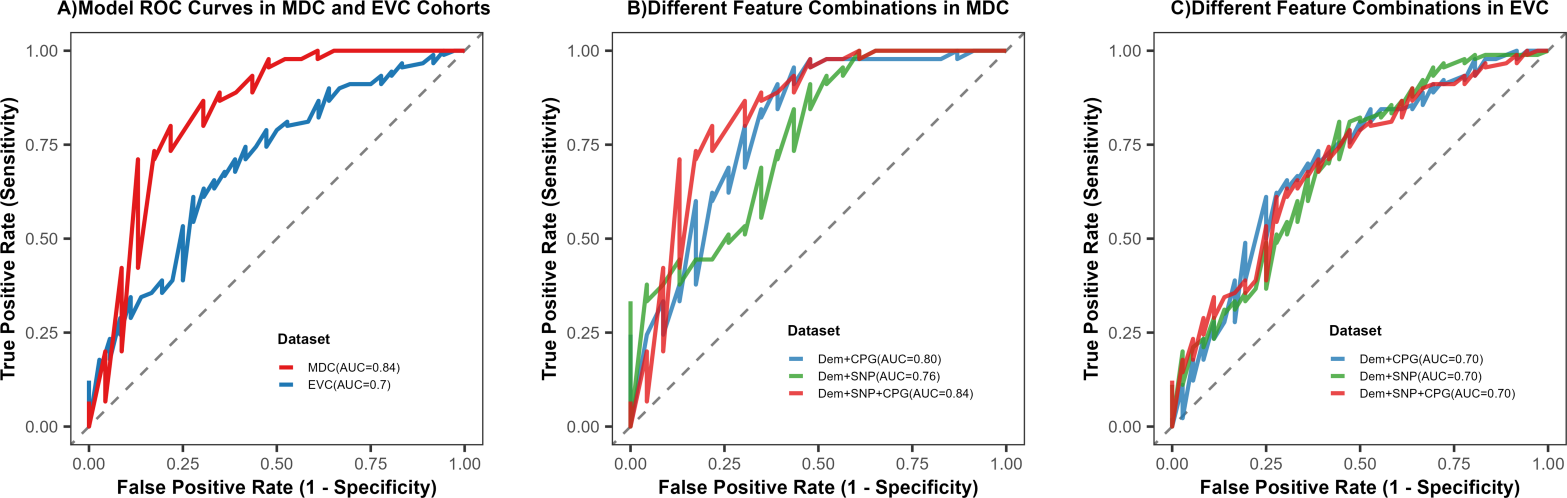
ROC analysis of the random forest LEF prediction model by cohort and feature set. **(A)** ROC curves and AUCs in MDC and EVC cohorts. **(B, C)** ROC curves and AUCs for different feature combinations in MDC and EVC.

In the independent EVC, the RF model maintained robust performance. The RF model achieves an AUC of 0.7 and an F1 Score of 0.84 on the EVC, verifying the generalization of the model in different patient populations. These results indicate that the integrated genetical-clinical model can reliably predict the LEF response in real-world clinical Settings (**Figure 6A**).

### 3.6 Performance comparison of models with different feature combinations across MDC and EVC cohorts

To compare model performance across datasets, we evaluated AUCs in the MDC and EVC (**Figures 6B, C**) using three predefined feature combinations: demographic variables plus SNPs only, CpGs only, or SNPs+CpGs. The RF model with Dem+SNPs+CpGs achieved optimal performance (AUC: 0.84 in MDC, 0.70 in EVC), outperforming the SNPs-only (AUC: 0.77, 0.70) and CpGs-only models (AUC: 0.80, 0.70) in both datasets.These results indicate that multi-omics integration improves predictive accuracy, particularly in the MDC cohort, while performance remained stable across models in the EVC cohort (all AUC: 0.70).

### 3.7 Functional enrichment, network analysis, and variant interaction​

**Figure 7:**
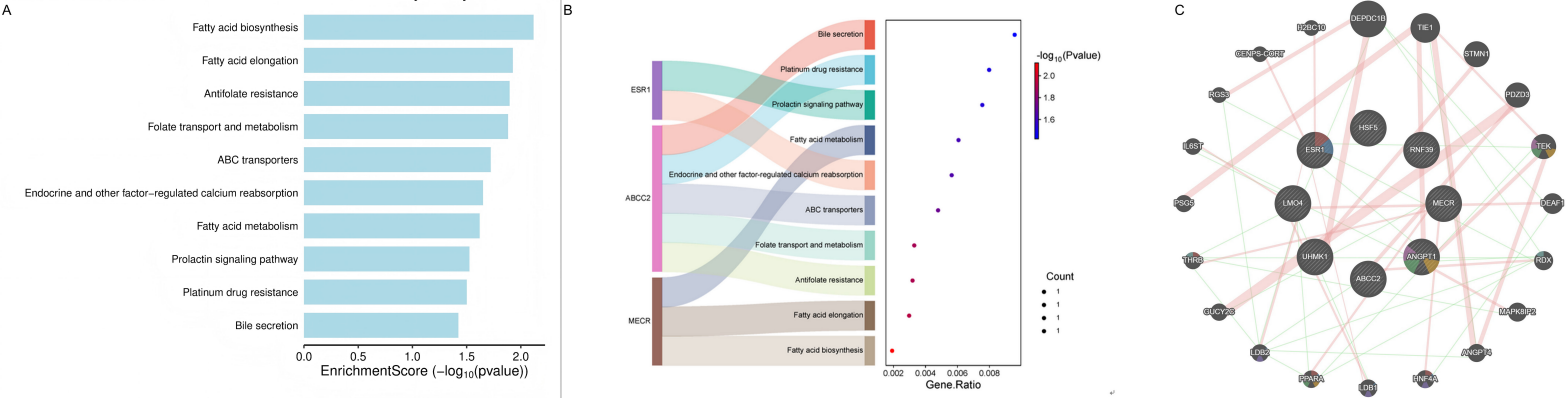
Functional enrichment, gene-pathway association, and protein-protein interaction network analyses of identified biomarkers. **(A)** Pathway enrichment bar plot showing −log_10_(P) values. **(B)** Circos plot integrating gene-pathway associations and enrichment results. **(C)** PPI network with functional annotation for key biomarkers.

To explore biological mechanisms underlying LEF response in RA, pathway enrichment analysis and gene network visualization were performed for the 10 genetic/epigenetic biomarkers (*ESR1, ABCC2, LMO4, MECR, ANGPT1, RNF39, UHMK1, HSF5*). As shown in **Figure 7A**, fatty acid biosynthesis was the most significantly enriched pathway (Enrichment Score=2.1), followed by fatty acid elongation, antifolate resistance, and folate transport/metabolism—highlighting the critical role of lipid metabolism and folate-related processes (key LEF targets) in mediating LEF response.

Complementary network analysis (**Figure 7B**) and **Supplementary Table 5** linked hub genes (*ESR1, ABCC2, MECR*) to pathways including ABC transporters (drug efflux), antifolate resistance (LEF’s core mechanism), and fatty acid metabolism (RA-related metabolic reprogramming). Functional association profiling via GeneMANIA further confirmed the eight core biomarkers are involved in ligand-activated transcription factor activity, transcription factor binding, thyroid hormone response, and epithelial cell apoptosis regulation. Detailed network analysis in **Figure 7C** revealed *ESR1* and *ANGPT1* form functional linkages via shared nodes: *ESR1* mediates transcriptional control (relevant to RA metabolic/immune regulation), while *ANGPT1* inhibits epithelial apoptosis—coordinating cell fate and tissue homeostasis. Given their dysregulation in inflammatory pathologies and inclusion in the LEF response model, we analyzed the interaction between cg07694252-*ANGPT1* and *ESR1*-rs2813563 using random forest framework, adjusted for baseline DAS28 and disease course in the MDC cohort.

**Figure 8:**
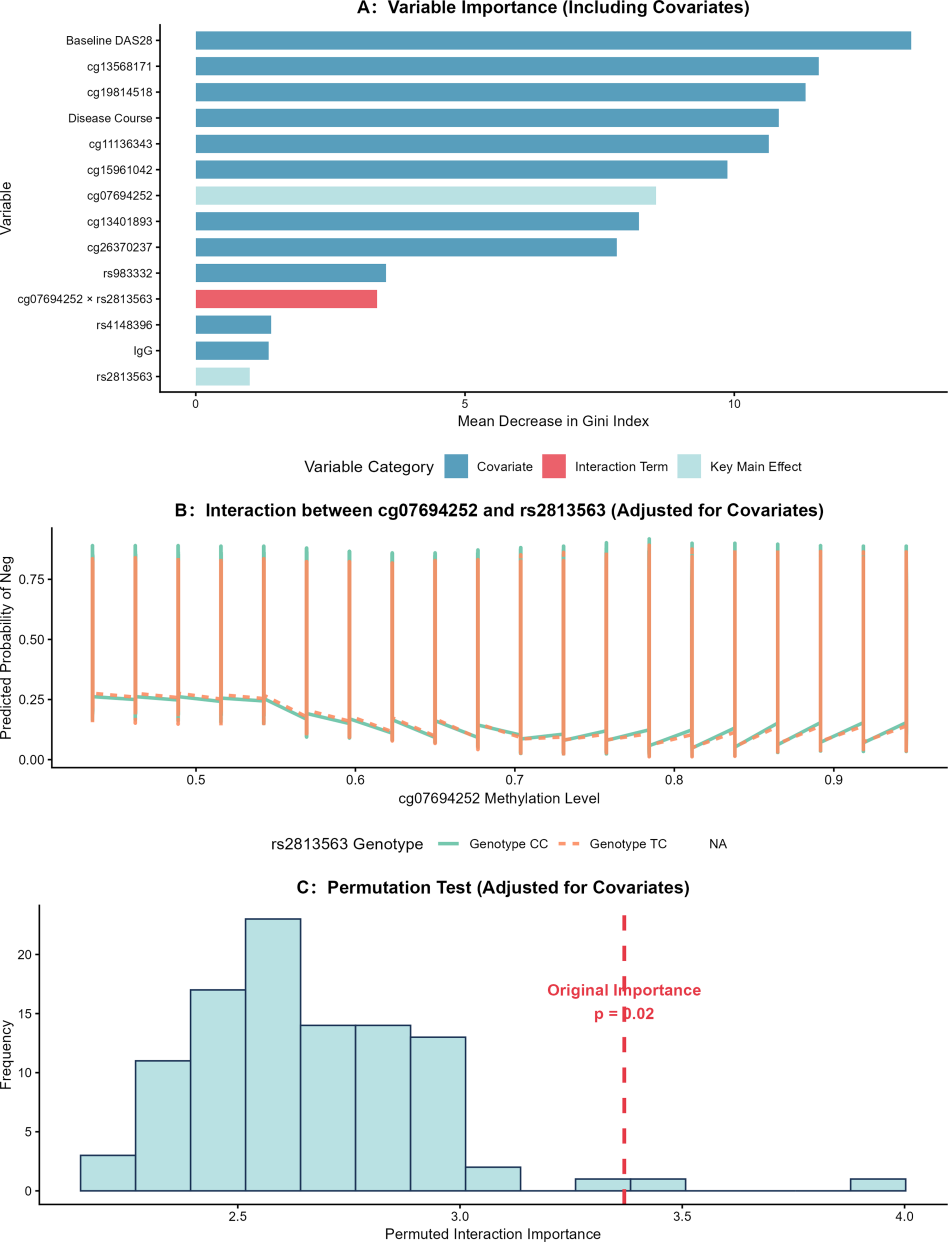
Integrated analysis of the interaction between cg07694252-*ANGPT1* and *ESR1*-rs2813563 in the MDC cohort. **(A)** Variable importance bar plot (including covariates). **(B)** Covariate-adjusted interaction effect of cg07694252-*ANGPT1* and *ESR1*-rs2813563. **(C)** Covariate-adjusted permutation test histogram (with original importance and p-value).

Variable importance assessment (**Figure 8A**) showed the interaction term (cg07694252-*ANGPT1*×*ESR1*-rs2813563) independently contributed to model performance (mean decrease in Gini index=3.37), exceeding ESR1-rs2813563’s main effect. Partial dependence plots (**Figure 8B**) demonstrated genotype-dependent methylation effects: at methylation<0.6, ESR1 CC/TC genotype predicted probabilities overlapped; at methylation>0.6, curves diverged (CC: downward-then-upward “Neg” outcome probability; TC: low and stable). A permutation test (**Figure 8C**) validated this interaction (*P* = 0.02).

## 4 Discussion

This multicenter, prospective cohort study addresses this unmet clinical need by developing an integrated ML-based predictive model that combines genetic, epigenetic, and clinical data—representing a significant advancement in precision medicine for RA. Our final model incorporates three novel SNPs (*ESR1*-rs2813563, *ABCC2*-rs4148396, *LMO4*-rs983332) and seven differentially methylated positions (DMPs:cg13568171-*MECR*, cg07694252-*ANGPT1*, cg13401893-*RNF39*, cg19814518-*UHMK1*, cg26370237-*HSF5*, cg11136343*-intergenic*, cg15961042-*intergenic*), alongside three clinical predictors (IgG, course of disease, baseline DAS28), achieving an AUC of 0.84 in the MDC and 0.70 in the EVC.

Building on our prior work that identified DNA methylation signatures as predictive of LEF response (11), the current study expands the analytical framework by: (1) increasing the sample size to enhance statistical power; (2) integrating SNPs with methylation data to capture multi-dimensional biological complexity; and (3) validating the model in an independent, geographically distinct external cohort. This integrative approach overcomes the limitations of single-omics studies, which often fail to account for the synergistic effects of genetic variation, epigenetic regulation, and clinical heterogeneity on treatment outcomes.

### 4.1 Key biological insights into LEF response mechanisms​

A major strength of this study is the identification of key predictive markers including *MECR, ABCC2*, and *ESR1*, which are enriched in pathways related to lipid metabolism, drug transport, and endocrine signaling—processes closely linked to the pharmacokinetic and pharmacodynamic profiles of LEF. These methylation markers represent baseline traits measured before treatment, rather than dynamic response-related changes. Notably, SHAP analysis highlighted cg13568171-*MECR* as the strongest predictive feature in both cohorts, and pathway analysis supported its involvement in lipid metabolism, supporting its role as a core biomarker for LEF response. However, all findings remain to be verified by experimental studies, and further functional investigations are required to clarify their exact biological roles.

### 4.2 Lipid metabolism: *MECR* as the pivotal biomarker linking epigenetics to LEF efficacy and toxicity​

*MECR*-mediated metabolism is critical for the proliferation and survival of CD4+ T cells, and may also reflect immune status in patients with mitochondrial fatty acid synthesis disorders, with potential implications for RA disease activity and prognosis (29, 30). Our results demonstrated that hypermethylation of cg13568171-*MECR* was associated with better clinical response to LEF. Mechanistically, *MECR* is closely linked to lipid metabolism and LEF-related liver safety: *MECR* dysfunction may modulate the synovial lipid microenvironment and inflammatory mediator production, while *MECR* silencing can disrupt hepatic fatty acid oxidation and exacerbate LEF-induced liver injury and dyslipidemia (30, 31). Notably, cg13568171-*MECR* serves not only as an efficacy predictor but also as a potential marker for LEF-related toxicity risk. Future studies are warranted to validate the association between cg13568171-*MECR* methylation and liver function (ALT, AST) or lipid profiles (TC, TG).

### 4.3 Drug metabolism and resistance: *ABCC2* as a core regulator of LEF bioavailability​

*ABCC2* and its rs4148396 polymorphism constitute another key biomarker, enriched in antifolate resistance, folate metabolism, and ABC transporter pathways—consistent with LEF’s role as a folate antagonist (32). *ABCC2* mediates drug efflux and modulates intracellular LEF levels, which govern DHODH inhibition in pyrimidine synthesis (33). SHAP analysis linked the *ABCC2*-rs4148396 variant to increased drug efflux and reduced response, in line with prior MTX studies in RA (34). This conserved mechanism across folate-related DMARDs supports *ABCC2* as a class-wide predictive marker, and high-risk patients may benefit from dose adjustment or ABC transporter inhibition.

### 4.4 Endocrine regulation: *ESR1* mediating gender-specific LEF response​

*ESR1* and its rs2813563 polymorphism were identified as key predictors by multi-omics integration, with pathway enrichment in endocrine resistance and calcium reabsorption. ESR1 participates in endocrine regulation and LEF metabolism: as a ligand-activated transcription factor, it modulates RA inflammation by inhibiting IL-6 and TNFα (35) and regulates LEF metabolism via CYP450 enzymes (36), which may contribute to gender-related differences in RA treatment response (37).

### 4.5 Other candidate biomarkers: underexplored pathways in LEF response​

While *LMO4*, *ANGPT1*, *RNF39*, *UHMK1*, and *HSF5* lacked significant pathway enrichment (likely due to limited database coverage or cell-type-specific expression), their inclusion in the final model—supported by univariate screening and LASSO dimensionality reduction—highlights their predictive value. Our multi-omics approach identifies these as underexplored regulators of LEF response: *LMO4* [a transcriptional regulator highly expressed in psoriatic epidermis (38, 39)] may modulate T cell differentiation in RA; *ANGPT1* [a key mediator of inflammatory angiogenesis (40)] could influence LEF’s tissue penetration by regulating synovial pannus formation; and *RNF39* [an innate immunity modulator (41)] may link infection and inflammation in RA. Single-cell RNA sequencing of RA synovial tissue—proposed as a future direction—could dissect their cell-type-specific regulatory networks and validate their functional relevance. And for the time being, there is no literature indicating the biological functions of cg11136343-*intergenic* and cg15961042-*intergenic*, which also requires further exploration.

### 4.6 Methylation-mediated regulation of *ESR1-ANGPT1* crosstalk: a functional switch for LEF treatment response

An exploratory mechanistic observation of the cg07694252-*ANGPT1*× *ESR1*-rs2813563 interaction in the development cohort points to a methylation-dependent functional window governing ESR1-mediated ANGPT1 regulation. At hypomethylation (<0.6), *ANGPT1* is transcriptionally silenced (42, 43), abrogating *ESR1* binding to its estrogen response element (44–46) and masking rs2813563 genotype-specific effects. Above this threshold, hypermethylation activates the locus, unmasking genotype-dependent differences: the CC genotype enables efficient *ANGPT1* upregulation [likely via intact ERE binding and Tie2/PI3K/Akt feedback (47–49)], while the TC genotype, with reduced *ESR1* affinity, shows persistently low activation. Thus, cg07694252-*ANGPT1* methylation may act as a molecular switch determining whether rs2813563 variation translates to LEF response phenotypes. However, this interaction was not replicated in the EVC, and the finding should therefore be regarded as hypothesis-generating rather than a definitive mechanistic conclusion, requiring further confirmation in larger cohorts.

### 4.7 Synergistic value of SNPs+CpGs dual genetic biomarkers over single biomarkers​

A critical innovation of this study is the integration of SNPs and CpG sites as dual biomarkers, which offers clear advantages over single-omic models. First, they provide complementary biological coverage: SNPs represent stable germline variation influencing drug transport and endocrine signaling, whereas CpG methylation reflects dynamic epigenetic regulation in lipid metabolism and immune pathways, jointly capturing both fixed genetic and plastic environmental components. Second, the integrated model achieved significantly improved performance in MDC (AUC = 0.84 vs 0.77/0.80) and equivalent robust performance in EVC (all AUC = 0.70), suggesting multi-omics integration enhances model stability and reproducibility without compromising predictive power in external settings.Third, dual markers enhance clinical robustness by reducing stratification bias and supporting refined four−tier risk stratification beyond binary high/low classification. Fourth, this integrative strategy clarifies gene−environment interplay and yields mechanistically interpretable signatures rather than purely predictive black−box markers.

### 4.8 Methodological advantages and clinical translation

This study employed rigorous methodology including multi-omics integration, ML optimization, and interpretable modeling to improve validity and translational potential. A structured feature selection pipeline retained only biologically meaningful predictors, and evaluation across ten ML algorithms reduced algorithm-specific bias and confirmed robustness. SHAP analysis quantified individual feature contributions and clarified context-dependent effects, mitigating the black-box nature of ML and improving clinical interpretability.

For clinical translation, our integrated model uses a small panel of key biomarkers and routine clinical variables to predict individual response to LEF in patients with RA, enabling early identification of non-responders and prompt adjustment to alternative DMARDs or combination therapy. The model relies on readily measurable markers without expensive or specialized equipment, supporting its feasibility for daily use in standard rheumatology settings as a decision-support tool for personalized LEF selection, rather than a general risk stratification instrument.

Sensitivity analyses employing different response definitions—including the stricter cutoff of ΔDAS28>1.2 for moderate to marked improvement, in contrast to ΔDAS28>0.6—further confirmed high stability of the core biomarkers across all thresholds.

Independent external validation in a geographically distinct cohort showed stable performance (AUC = 0.70), suggesting favorable generalizability. Although this AUC is modest for conclusive clinical decision-making, it provides a meaningful real-world signal for pretreatment identification of patients likely to benefit from LEF. In clinical practice with multiple DMARD options, this predictive tool can help personalize early treatment selection and streamline therapeutic management.

### 4.9 Limitations and future directions​

Despite its strengths, this study has several limitations. First, the modest sample size may cause mild overfitting, and validation limited to Chinese Han participants restricts generalizability; findings are exploratory and require validation in larger, more diverse cohorts. Second, residual confounding may exist from unadjusted factors (e.g., concomitant medications, treatment adherence, comorbidities). Third, the cg07694252-*ANGPT1*×*ESR1*-rs2813563 interaction identified in the development cohort was not replicated in external validation (*P* = 0.68) and is hypothesis-generating only. Fourth, functional validation of biomarkers and the epigenetic mechanisms of DMPs are lacking. Fifth, only 6-month LEF response was evaluated, with no prospective testing of long-term outcomes (e.g., sustained remission, joint damage). Sixth, the long-term stability of methylation markers is undetermined, and no formal batch effect correction was performed for omics data, which may introduce subtle technical biases. Future studies should verify biological causality via multi-omics and experiments, conduct prospective trials to evaluate model-guided outcomes, and facilitate clinical translation for real-world use.

## 5 Conclusions​

This study advances precision medicine for RA by developing and validating an integrated ML-based model that predicts LEF response using multi-dimensional data. The identification of novel genetic/epigenetic biomarkers and their mechanistic characterization into three functional modules (lipid metabolism, drug transport, endocrine signaling) provides new insights into LEF’s therapeutic action. Methodological rigor—including multi-algorithm optimization, SHAP-driven interpretability, and external validation—ensures the model’s reliability and clinical feasibility. This study provides a promising multi-omics predictive model for LEF response in RA. The current model remains at the research stage; larger, prospective, and ethnically diverse validation studies are essential before consideration for routine clinical implementation. Our findings support the value of multi-omics integration for future precision rheumatology.

## Data Availability

The raw multi-omics data supporting the findings of this study have been deposited in the OMIX (Omics Archive) database of the National Genomics Data Center (NGDC), China National Center for Bioinformation (CNCB), under accession number OMIX017508. The data are publicly available and can be accessed here: https://ngdc.cncb.ac.cn/omix/release/OMIX017508.

## Supplementary material

Supplementary Figure 1 Comprehensive workflow for SNP screening integrating WES analysis, BIOINFORMATICS TECHNOLOGIES, AND VALIDATION TESTING.

Supplementary Table 1 Classification criteria for biochemical, immunological, glucose-lipid metabolism and hematological indicators.

Supplementary Table 2 center-specific classification standards for biochemical-immunological, glucose-lipid metabolism and routine blood indicators.

Supplementary Table 3 Demographic and clinical characteristics of the study population, stratified by cohort and group status.

Supplementary Table 4 performance comparison of ten ML models for LEF prognostic prediction across key diagnostic metrics in the MDC dataset.

Supplementary Table 5 Genetic markers identified for constructing the leflunomide prognostic model.

Supplementary Table 6 Distribution of disease activity status according to DAS28 criteria among 365 patients with rheumatoid arthritis.
