# Supplementary material for: Integrating genetic, epigenetic, and clinical signatures via machine learning for robust prediction of leflunomide response in rheumatoid arthritis: a multi-center validation study
Source: Front Immunol. 2026 Jun 24;17:1804485. doi: 10.3389/fimmu.2026.1804485 (PMC13342399; doi:10.3389/fimmu.2026.1804485)
Supplement: Supplementary Figure 1 — Comprehensive workflow for SNP screening integrating WES analysis, BIOINFORMATICS TECHNOLOGIES, AND VALIDATION TESTING. [file SupplementaryFile1.docx]

**
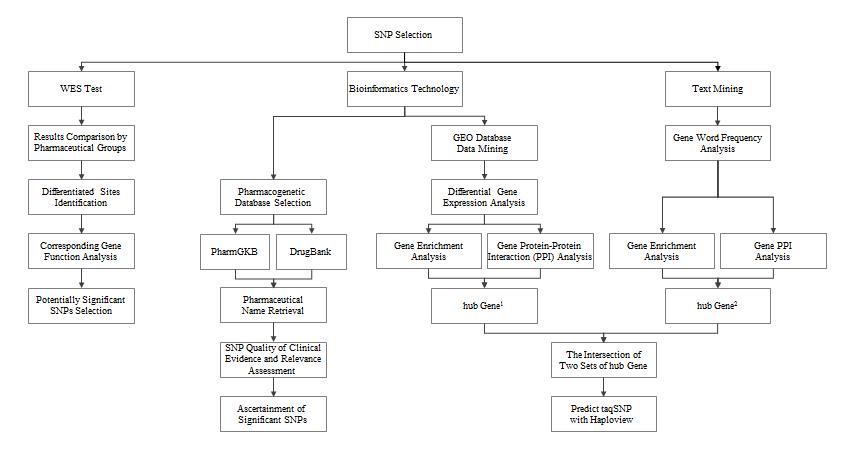
**

Supplemental Figure 1: Comprehensive Workflow for SNP Screening Integrating WES Analysis, Bioinformatics Technologies, and Validation Testing
