# Supplementary material for: Integrating genetic, epigenetic, and clinical signatures via machine learning for robust prediction of leflunomide response in rheumatoid arthritis: a multi-center validation study
Source: Front Immunol. 2026 Jun 24;17:1804485. doi: 10.3389/fimmu.2026.1804485 (PMC13342399; doi:10.3389/fimmu.2026.1804485)
Supplement: Supplementary Table 1 — Classification criteria for biochemical, immunological, glucose-lipid metabolism and hematological indicators. [file Table1.docx]

Supplemental Table 1: Classification Criteria for Biochemical, Immunological, Glucose-Lipid Metabolism and Hematological Indicators

| Research center | The First Affiliated Hospital of China Medical University | | Shengjing Hospital affiliated to China Medical University | | The First Affiliated Hospital of Jinzhou Medical University | | Dalian Central Hospital | |
| --- | --- | --- | --- | --- | --- | --- | --- | --- |
|  | Low | high | Low | high | Low | high | Low | high |
| NE（*10^9/L） | ≤6.3 | >6.3 | ≤7.2 | >7.2 | ≤6.3 | >6.3 | ≤6.3 | >6.3 |
| PCT（L/L） | ≤0.3 | >0.3 | ≤0.28 | >0.28 | ≤0.36 | >0.36 | ≤0.36 | >0.36 |
| Anti-CCP（U/ml） | ≤17 | >17 | ≤20 | >20 | ≤2.5 | >2.5 | ≤5.00 | >5.00 |
| RF（IU/ml） | ≤20 | >20 | ≤20 | >20 | ≤15.9 | >15.9 | ≤20 | >20 |
| IgG（g/L） | ≤17 | >17 | ≤16 | >16 | ≤16 | >16 | ≤16 | >16 |
| IgM（g/L） | ≤2.5 | >2.5 | ≤2.3 | >2.3 | ≤2.3 | >2.3 | ≤2.3 | >2.3 |
| IgA（g/L） | ≤3.8 | >3.8 | ≤4.0 | >4.0 | ≤4.0 | >4.0 | ≤4.0 | >4.0 |
| C3（g/L） | ≤1.5 | >1.5 | ≤1.8 | >1.8 | ≤1.8 | >1.8 | ≤1.8 | >1.8 |
| C4（g/L） | ≤0.36 | >0.36 | ≤0.4 | >0.4 | ≤0.4 | >0.4 | ≤0.4 | >0.4 |
| LD（mmol/L） | ≤4.1 | >4.1 | ≤3.37 | >3.37 | ≤3.63 | >3.63 | ≤3.12 | >3.12 |
| HDL（mmol/L） | <0.7 | ≥0.7 | <1.04 | ≥1.04 | ≤1.04 | >1.04 | <1.04 | ≥1.04 |
| TC（mml/L） | ≤5.5 | >5.5 | ≤5.69 | >5.69 | ≤5.7 | >5.7 | ≤5.18 | >5.18 |
| TG（mml/L） | ≤1.69 | >1.69 | ≤1.69 | >1.69 | ≤1.70 | >1.70 | ≤1.70 | >1.70 |
| GLU（mmol/L） | ≤6.1 | >6.1 | ≤6.11 | >6.11 | ≤6.11 | >6.11 | ≤6.10 | >6.10 |
| CRP（mg/L） | ≤5 | >5 | ≤8 | >8 | ≤1 | >1 | ≤8 | >8 |
| ESR（mm/h） | ≤20 | >20 | ≤20 | >20 | ≤26 | >26 | ≤30 | >30 |
| MONO（*10^9/L） | ≤ 0.6 | >0.6 | ≤ 0.8 | >0.8 | ≤ 0.6 | >0.6 | ≤ 0.6 | >0.6 |
| PLT（*10^9/L） | ≤350 | >350 | ≤ 350 | >350 | ≤ 350 | >350 | ≤ 350 | >350 |
