# Supplementary material for: Integrating genetic, epigenetic, and clinical signatures via machine learning for robust prediction of leflunomide response in rheumatoid arthritis: a multi-center validation study
Source: Front Immunol. 2026 Jun 24;17:1804485. doi: 10.3389/fimmu.2026.1804485 (PMC13342399; doi:10.3389/fimmu.2026.1804485)
Supplement: Supplementary Table 2 — center-specific classification standards for biochemical-immunological, glucose-lipid metabolism and routine blood indicators. [file Table2.docx]

Supplemental Table 2: Center-Specific Classification Standards for Biochemical-Immunological, Glucose-Lipid Metabolism and Routine Blood Indicators

| Research center | The First Affiliated Hospital of China Medical University | | | Shengjing Hospital affiliated to China Medical University | | | The First Affiliated Hospital of Jinzhou Medical University | | | Dalian Central Hospital | | |
| --- | --- | --- | --- | --- | --- | --- | --- | --- | --- | --- | --- | --- |
|  | Low | Mid | high | Low | Mid | high | Low | Mid | high | Low | Mid | high |
| LY（*10^9/L） | <1.1 | ≥1.1and≤3.2 | >3.2 | <1.1 | ≥1.1and≤2.7 | >2.7 | <1.1 | ≥1.1and≤3.2 | >3.2 | <1.1 | ≥1.1and≤3.2 | >3.2 |
| PDW（%） | <9.4 | ≥9.4and≤16.0 | >16.0 | <11.5 | ≥11.5and≤16.5 | >16.5 | <9.3 | >9.3and≤17.0 | >17.0 | <9.0 | ≥9.0and≤17.0 | >17.0 |
| RB（*10^12/L） | <3.8 | ≥3.8and≤5.1 | >5.1 | <3.68 | ≥3.68and≤5.13 | >5.13 | <3.8 | ≥3.8and≤5.1 | >5.1 | <3.8 | ≥3.8and≤5.1 | >5.1 |
| HGB（g/L） | <115 | ≥115and≤150 | >150 | <110 | ≥110and≤150 | >150 | <115 | ≥115and≤150 | >150 | <115 | ≥115and≤150 | >150 |
