# Supplementary material for: Integrating genetic, epigenetic, and clinical signatures via machine learning for robust prediction of leflunomide response in rheumatoid arthritis: a multi-center validation study
Source: Front Immunol. 2026 Jun 24;17:1804485. doi: 10.3389/fimmu.2026.1804485 (PMC13342399; doi:10.3389/fimmu.2026.1804485)
Supplement: Supplementary Table 3 — Demographic and clinical characteristics of the study population, stratified by cohort and group status. [file Table3.docx]

Supplemental Table 3: Demographic and Clinical Characteristics of the Study Population, Stratified by Cohort and Group Status

| Variable | | MDC Cohort (n=231) | | | EVC Cohort (n=126) | | | *P* Value (MDC vs. EVC) |
| --- | --- | --- | --- | --- | --- | --- | --- | --- |
|  |  | MDC-non-responders  (n=78) | MDC-responders (n=153) | P Value | EVC-non-responders  (n=36) | EVC-responders (n=90) | *P* Value |  |
| Age | | 59.4 ± 13.6 | 59.0 ± 13.3 | 0.79 | 56.7 ± 10.2 | 58.4 ± 10.1 | 0.31 | 0.19 |
| Course of Disease | | 9.7 ± 9.6 | 5.3 ± 7.5 | <0.01 | 7.3 ± 8.4 | 3.2 ± 4.5 | <0.01 | 0.03 |
| Baseline DAS28 | | 5.5 ± 1.8 | 6.2 ± 1.3 | <0.01 | 4.0 ± 1.8 | 5.4 ± 1.4 | <0.01 | <0.01 |
| Gender, n (%) | Male | 19 (24.4) | 37 (24.2) | 1.00 | 8 (22.2) | 15 (16.7) | 0.64 | 0.24 |
|  | Female | 59 (75.6) | 116 (75.8) | - | 28 (77.8) | 75 (83.3) | - |  |
| Anti-CCP,  n (%) | Low level | 15 (19.2) | 34 (22.2) | 0.72 | 10 (27.8) | 7 (7.8) | 0.01 | 0.10 |
|  | High level | 63 (80.8) | 119 (77.8) | - | 26 (72.2) | 83 (92.2) | - |  |
| RF,  n (%) | Low level | 19 (24.4) | 47 (30.7) | 0.39 | 8 (22.2) | 15 (16.7) | 0.64 | 0.04 |
|  | High level | 59 (75.6) | 106 (69.3) | - | 28 (77.8) | 75 (83.3) | - |  |
| IgG,  n (%) | Low level | 69 (88.5) | 118 (77.1) | 0.06 | 31 (86.1) | 67 (74.4) | 0.24 | 0.56 |
|  | High level | 9 (11.5) | 35 (22.9) | - | 5 (13.9) | 23 (25.6) | - |  |
| Diabetes, n (%) | No | 72 (92.3) | 138 (90.2) | 0.78 | 35 (97.2) | 81 (90.0) | 0.28 | 0.86 |
|  | Yes | 6 (7.7) | 15 (9.8) | - | 1 (2.8) | 9 (10.0) | - |  |
| Hypertension,  n (%) | No | 66 (84.6) | 126 (82.4) | 0.80 | 31 (86.1) | 75 (83.3) | 0.91 | 0.92 |
|  | Yes | 12 (15.4) | 27 (17.6) | - | 5 (13.9) | 15 (16.7) | - |  |
| ESR,  n (%) | Low level | 24 (30.8) | 31 (20.3) | 0.11 | 18 (50.0) | 23 (25.6) | 0.02 | 0.10 |
|  | High level | 54 (69.2) | 122 (79.7) | - | 18 (50.0) | 67 (74.4) | - |  |
