# Supplementary material for: Integrating genetic, epigenetic, and clinical signatures via machine learning for robust prediction of leflunomide response in rheumatoid arthritis: a multi-center validation study
Source: Front Immunol. 2026 Jun 24;17:1804485. doi: 10.3389/fimmu.2026.1804485 (PMC13342399; doi:10.3389/fimmu.2026.1804485)
Supplement: Supplementary Table 4 — performance comparison of ten ML models for LEF prognostic prediction across key diagnostic metrics in the MDC dataset. [file Table4.docx]

Supplemental Table 4: Performance Comparison of Ten ML Models for LEF Prognostic Prediction Across Key Diagnostic Metrics in the MDC Dataset

| Model | Accuracy | Sensitivity | Specificity | PPV | NPV | F1 | AUC |
| --- | --- | --- | --- | --- | --- | --- | --- |
| Random Forest | 0.82 (0.71-0.91) | 0.98 (0.88-1.00) | 0.52 (0.31-0.73) | 0.80 (0.67-0.90) | 0.92 (0.64-1.00) | 0.88 (0.81-0.94) | 0.84 (0.73-0.94) |
| Gradient Boosting | 0.75 (0.63-0.85) | 0.82 (0.68-0.92) | 0.61 (0.39-0.80) | 0.80 (0.66-0.91) | 0.64 (0.41-0.83) | 0.81 (0.72-0.89) | 0.83 (0.73-0.92) |
| SVM (Radial Kernel) | 0.71 (0.58-0.81) | 0.78 (0.63-0.89) | 0.57 (0.35-0.77) | 0.78 (0.63-0.89) | 0.57 (0.35-0.77) | 0.78 (0.67-0.86) | 0.78 (0.65-0.88) |
| Logistic Regression | 0.75 (0.63-0.85) | 0.82 (0.68-0.92) | 0.61 (0.39-0.80) | 0.80 (0.66-0.91) | 0.64 (0.41-0.83) | 0.81 (0.72-0.89) | 0.79 (0.66-0.89) |
| K-Nearest Neighbors | 0.68 (0.55-0.79) | 0.82 (0.68-0.92) | 0.39 (0.20-0.62) | 0.73 (0.58-0.84) | 0.53 (0.28-0.77) | 0.77 (0.67-0.85) | 0.63 (0.50-0.76) |
| Partial Least Squares | 0.74 (0.61-0.84) | 0.84 (0.71-0.94) | 0.52 (0.31-0.73) | 0.78 (0.63-0.88) | 0.63 (0.38-0.84) | 0.81 (0.71-0.89) | 0.77 (0.63-0.88) |
| AdaBoost | 0.74 (0.61-0.84) | 0.96 (0.85-1.00) | 0.30 (0.13-0.53) | 0.73 (0.60-0.84) | 0.78 (0.40-0.97) | 0.83 (0.74-0.90) | 0.77 (0.65-0.88) |
| Naive Bayes | 0.71 (0.58-0.81) | 0.80 (0.65-0.90) | 0.52 (0.31-0.73) | 0.77 (0.62-0.88) | 0.57 (0.34-0.78) | 0.78 (0.68-0.87) | 0.75 (0.62-0.86) |
| Linear Discriminant Analysis | 0.71 (0.58-0.81) | 0.80 (0.65-0.90) | 0.52 (0.31-0.73) | 0.77 (0.62-0.88) | 0.57 (0.34-0.78) | 0.78 (0.68-0.87) | 0.78 (0.66-0.89) |
| Lasso Regression | 0.75 (0.63-0.85) | 0.82 (0.68-0.92) | 0.61 (0.39-0.80) | 0.80 (0.66-0.91) | 0.64 (0.41-0.83) | 0.81 (0.72-0.89) | 0.79 (0.66-0.89) |
