# Supplementary material for: Integrating genetic, epigenetic, and clinical signatures via machine learning for robust prediction of leflunomide response in rheumatoid arthritis: a multi-center validation study
Source: Front Immunol. 2026 Jun 24;17:1804485. doi: 10.3389/fimmu.2026.1804485 (PMC13342399; doi:10.3389/fimmu.2026.1804485)
Supplement: Supplementary Table 5 — Genetic markers identified for constructing the leflunomide prognostic model. [file Table5.docx]

Supplemental Table 5: Genetic Markers Identified for Constructing the Leflunomide Prognostic Model

| SNP/CPG | gene | Chromosome | location | function |
| --- | --- | --- | --- | --- |
| rs2813563 | ESR1 | 6 | chr6:152125352 | Estrogen receptor α; regulates cell proliferation,differentiation, and hormone-dependent cancers |
| rs4148396 | ABCC2 | 10 | chr10:99832187 | ATP-binding cassette transporter; mediates drug excretion and chemoresistance. |
| rs983332 | LMO4 | 1 | chr1:87666697 | LIM domain protein; modulates transcription, cell cycle, and breast cancer progression |
| cg07694252 | ANGPT1 | 8 | chr8:107,428,726-107,428,727 | Angiopoietin-1; promotes vascular maturation, endothelial survival, and angiogenesis. |
| cg11136343 | - | 5 | chr5:163,846,696-163,846,697 | Intergenic CpG; no annotated protein-coding gene; may regulate distal gene expression |
| cg13401893 | RNF39 | 6 | chr6:30,071,655-30,071,656 | E3 ubiquitin ligase; involved in innate immunity, antiviral signaling, and synaptic plasticity. |
| cg13568171 | MECR | 1 | chr1:29,230,309-29,230,310 | Mitochondrial enoyl-CoA reductase; catalyzes mtFAS final step; essential for lipoic acid synthesis. |
| cg15961042 | - | 12 | chr12:38,055,154-38,055,155 | Intergenic CpG; no annotated protein-coding gene; potential long-range regulatory element |
| cg19814518 | UHMK1 | 1 | chr1:162,497,290-162,497,291 | Ser/Thr kinase; regulates cell cycle, RNA processing, and neuronal function. |
| cg26370237 | HSF5 | 17 | chr17:58,436,870-58,436,871 | Heat shock transcription factor 5; critical for spermatogenesis and male meiosis progression. |
