# Supplementary material for: Integrating genetic, epigenetic, and clinical signatures via machine learning for robust prediction of leflunomide response in rheumatoid arthritis: a multi-center validation study
Source: Front Immunol. 2026 Jun 24;17:1804485. doi: 10.3389/fimmu.2026.1804485 (PMC13342399; doi:10.3389/fimmu.2026.1804485)
Supplement: Supplementary Table 6 — Distribution of disease activity status according to DAS28 criteria among 365 patients with rheumatoid arthritis. [file Table6.docx]

Supplemental Table 6 Distribution of disease activity status according to DAS28 criteria among 365 patients with rheumatoid arthritis

| Status (Abbreviation) | DAS28 Cutoff | Patients (n=357) | Proportion (%) |
| --- | --- | --- | --- |
| Remission | < 2.6 | 83 | 23.25% |
| Low Disease Activity (LDA) | 2.6 ≤ DAS28 < 3.2 | 47 | 13.17% |
| Moderate Disease Activity (MDA) | 3.2 ≤ DAS28 < 5.1 | 128 | 35.85% |
| High Disease Activity (HDA) | ≥ 5.1 | 99 | 27.73% |
